# A Novel Non-Coding Variant in DCLRE1C Results in Deregulated Splicing and Induces SCID Through the Generation of a Truncated ARTEMIS Protein That Fails to Support V(D)J Recombination and DNA Damage Repair

**DOI:** 10.3389/fimmu.2021.674226

**Published:** 2021-06-17

**Authors:** Steven Strubbe, Marieke De Bruyne, Ulrich Pannicke, Elien Beyls, Bart Vandekerckhove, Georges Leclercq, Elfride De Baere, Victoria Bordon, Anne Vral, Klaus Schwarz, Filomeen Haerynck, Tom Taghon

**Affiliations:** ^1^ Department of Diagnostic Sciences, Ghent University, Ghent, Belgium; ^2^ Center for Medical Genetics Ghent (CMGG), Ghent, Belgium; ^3^ The Institute for Transfusion Medicine, University of Ulm, Ulm, Germany; ^4^ Department of Human Structure and Repair, Ghent University, Ghent, Belgium; ^5^ Cancer Research Institute Ghent (CRIG), Ghent, Belgium; ^6^ Department of Biomolecular Medicine, Ghent University, Ghent, Belgium; ^7^ Department of Internal Medicine and Pediatrics, Division of Pediatric Hemato-Oncology and Stem Cell Transplantation, Ghent University Hospital, Ghent, Belgium; ^8^ Institute for Clinical Transfusion Medicine and Immunogenetics Ulm, Germa Red Cross Blood Service Baden-Württemberg – Hessen, Ulm, Germany; ^9^ Primary Immunodeficiency Research Lab, Jeffrey Modell Diagnosis and Research Center, Ghent University Hospital, Ghent, Belgium; ^10^ Department of Internal Medicine and Pediatrics, Division of Pediatric Immunology and Pulmonology, Ghent University Hospital, Ghent, Belgium

**Keywords:** SCID, NGS, ARTEMIS, V(D)J recombination, DNA damage repair

## Abstract

Severe Combined Immune Deficiency (SCID) is a primary deficiency of the immune system in which opportunistic and recurring infections are often fatal during neonatal or infant life. SCID is caused by an increasing number of genetic defects that induce an abrogation of T lymphocyte development or function in which B and NK cells might be affected as well. Because of the increased availability and usage of next-generation sequencing (NGS), many novel variants in SCID genes are being identified and cause a heterogeneous disease spectrum. However, the molecular and functional implications of these new variants, of which some are non-coding, are often not characterized in detail. Using targeted NGS, we identified a novel homozygous c.465-1G>C splice acceptor site variant in the *DCLRE1C* gene in a T^-^B^-^NK^+^ SCID patient and fully characterized the molecular and functional impact. By performing a minigene splicing reporter assay, we revealed deregulated splicing of the *DCLRE1C* transcript since a cryptic splice acceptor in exon 7 was employed. This induced a frameshift and the generation of a p.Arg155Serfs*15 premature termination codon (PTC) within all *DCLRE1C* splice variants, resulting in the absence of full-length ARTEMIS protein. Consistently, a V(D)J recombination assay and a G0 micronucleus assay demonstrated the inability of the predicted mutant ARTEMIS protein to perform V(D)J recombination and DNA damage repair, respectively. Together, these experiments molecularly and functionally clarify how a newly identified c.465-1G>C variant in the *DCLRE1C* gene is responsible for inducing SCID. In a clinical context, this demonstrates how the experimental validation of new gene variants, that are identified by NGS, can facilitate the diagnosis of SCID which can be vital for implementing appropriate therapies.

## Introduction

Severe Combined Immune Deficiency (SCID), a subgroup of Primary Immune Deficiency (PID), has an estimated incidence of 1 in 50,000-100,000 livebirths and is caused by rare genetic defects which abrogate the normal development and/or function of T lymphocytes while B and Natural Killer (NK) cells can be affected as well (1, 2). SCID leads, amongst other symptoms, to increased susceptibility to infections during the first few months of life with failure to thrive and results in early morbidity and mortality in the most severe cases. Over the last decade, next-generation sequencing (NGS) technologies permitted the identification of an increasing number of genes implicated in SCID, with currently more than 20 SCID-associated genes of which multiple variants exist (3–5). In addition, newborn screening (NBS) of SCID is now implemented as standard care in several countries (e.g. USA, Germany) since this approach has shown to be effective to identify SCID before the onset of disease (6–8). Furthermore, NBS more accurately defines the distribution of different SCID genotypes since neonates are now screened before infections can become fatal (9). This wide variety of immunological subtypes, caused by a continuously increasing repertoire of SCID-associated genes and variants, results in a highly heterogeneous spectrum of SCID diseases (9). Hypomorphic mutations, categorized as ‘leaky SCID’, cause a milder phenotype with often a later onset of disease and also contribute to the complexity of these distinct immunological phenotypes (10–15). Currently, allogeneic hematopoietic stem cell transplantation (HSCT) remains the treatment of choice but the genetic subtype greatly impacts long-term survival and immune reconstitution (16). Therefore, for some SCID genetic defects, gene therapy studies using autologous stem cells seem to be promising as an alternative approach to cure disease as illustrated for ADA-, IL2RG- and ARTEMIS-SCID (17–21).

V(D)J recombination is a hallmark of both T and B cell development and is responsible for the diversity of immune responses mediated by T and B lymphocytes. Both T cell receptor (TCR) and Immunoglobulin (Ig) gene loci consist of multiple germline Variable (V), Diversity (D) and Joining (J) gene segments that are recombined through a strictly regulated process to generate in-frame functional antigen receptors. Eventually, this mechanism ensures the generation of a highly diverse repertoire of polyclonal T and B cells, all characterized by a unique TCR and B cell receptor (BCR) respectively (22). Pathogenic mutations in genes that encode for proteins implicated in V(D)J recombination (23), such as mutations in *RAG1*) and *RAG2* (24), *DCLRE1C* (11, 25), *PRKDC* (26, 27) and *LIG4* (28, 29), typically cause T^-^B^-^NK^+^ SCID and together account for approximately 20-25% of all SCID. *RAG1* and *RAG2* mutations make up the large majority of these whereas *DCLRE1C* mutations for instance, of which the founder variant was first characterized in Athabascan-speaking Navajo Native Americans, account for only around 20% of T^-^B^-^NK^+^ SCID and thus 3-4% of all SCID cases (9, 30). Initially, V(D)J recombination is initiated by the RAG proteins that cooperate to induce DNA double-stranded breaks (DSBs) in between the germline V, D and J segments to ultimately form a hairpin coding end and a blunt signal end. Next, binding of the KU70/80 complex to these DSBs is required to activate the DNA-dependent protein kinase catalytic subunit (DNA-PKcs), encoded by the *PRKDC* gene. This protein then in turn phosphorylates the ARTEMIS protein, encoded by *DCLRE1C*, to activate its nuclease activity which is important for cleavage of the hairpin coding end (31). Eventually, the XRCC4/LIG4 complex, in the presence of XLF/CERNUNNOS, catalyzes the non-homologous end-joining (NHEJ) of these cleaved coding gene segments in order to assemble a successfully recombined allele. Aberrations in any of these processes therefore lead to a strong reduction of mature T and B cells or to a strongly reduced diversity in the antigen receptor repertoire of these lymphocytes, thereby causing SCID (23). V(D)J recombination might also be implicated in NK cell development since immunoregulatory CD56^bright^CD16^-/int^NKG2A^+++^ NK cells are increased in the peripheral blood of V(D)J recombination-deficient SCID patients at the expense of cytotoxic CD56^dim^CD16^hi^ NK cells (32). Moreover, NK cells from V(D)J recombination-deficient SCID patients display an enhanced effector function, similar to NK cells from *Rag*-deficient mice (33). Whether CD56^dim^ NK lymphocytes arise from CD56^bright^ NK cells, or whether both subsets have distinct developmental origins is not entirely clear (34–36).

Using NGS, we identified a novel non-coding homozygous c.465-1G>C variant of the *DCLRE1C* gene in a patient who was characterized by a severe T^-^B^-^NK^+^ SCID phenotype. This variant affects the splice acceptor site of intron 6 and causes a frameshift that ultimately results in a premature termination codon (PTC). Functional assays to study the residual activity of the predicted truncated ARTEMIS protein reveal a severe reduction in both V(D)J recombination and DNA damage repair which can be directly linked to the phenotype of this patient. Our work thus clarifies at the molecular level how this novel genetic variant in the *DCLRE1C* gene causes SCID. Furthermore, these experimental validations highlight the importance of investigating the molecular pathogenic mechanisms of new variants in SCID genes, including non-coding, since *in silico* predictions tools remain largely insufficient to estimate their pathogenicity (37).

## Materials and Methods

### Flow Cytometry of Peripheral Blood Mononuclear Cells (PBMCs)

All human samples were obtained and used according to the guidelines of the Medical Ethical Commission of Ghent University Hospital (Belgium). The age of the six healthy children, used as controls, ranged from 7-years-old to 14-years-old. First, PBMCs were isolated using a Lymphoprep density gradient (Elitech) and a fraction of these cells was washed and resuspended in MACS buffer (PBS, 2% FCS, 2mM EDTA) before staining. Second, non-specific binding was prevented by blocking of all Fc receptors in a 10-minute incubation step with human FcR blocking reagent (Miltenyi). Next, cells were stained for 30 minutes at 4°C and protected from light, using the following anti-human antibodies according to the manufacturer’s instructions: CD3-APCCy7 (Biolegend, clone UCHT1), CD4-PECy7 (Biolegend, clone OKT4), CD8β-PE (Beckman Coulter, clone 2ST8.5H7), CD14-APC (Biolegend, clone M5E2), CD14-PE (Biolegend, clone M5E2) CD19-FITC (Biolegend, clone HIB19), CD45-PerCPCy5.5 (Biolegend, clone HI30), CD56-APC (Biolegend, clone 5.1H11) and HLA-DR-APCCy7 (ThermoFisher Scientific, clone LN3). After staining, cells were washed and resuspended in human washing buffer (PBS, 1% BSA, 0.1% NaN_3_). Immediately before measurement on the LSRII flow cytometer (BD), propidium iodide was added to exclude dead cells. Analysis of flow cytometry data was performed using the FlowJo™ Software, version 10.7.1 for Windows (BD).

### Genetic Analyses on Genomic DNA

Targeted NGS of genes associated with SCID was performed in the index patient. Genomic DNA was isolated from whole blood cells according to standard procedures. Primers for amplification and sequencing of the coding regions and adjacent intron-exon boundaries of *DCLRE1C, LIG4, NHEJ1, PRKDC, RAG1, RAG2* and *ZAP70* were designed using an in-house developed primer design software program (www.pxlence.com). Targeted NGS was performed using a flexible protocol, consisting of singleplex PCR followed by NexteraXT library preparation and sequencing on a MiSeq instrument as previously described (38). The CLC Genomics Workbench v.6 (Qiagen) was employed for read mapping against the hg19 human reference genome and variant calling. For subsequent data analysis, variants were filtered based on technical quality parameters, frequency in public population databases and *in silico* predictions. More thorough variant annotation of the remaining variants was executed using Alamut Visual software (Interactive Biosoftware) and a complementary literature study. Sanger sequencing was used to confirm the causal *DCLRE1C* variant that was identified by NGS and to perform segregation analysis in all available family members. Genomic DNA was amplified by PCR using the specific primers and KAPA2G Robust Hotstart Ready Mix (KAPA Biosystems). PCR products were enzymatically purified with Exonuclease I and Antarctic phosphatase (both New England BioLabs, NEB). Purified PCR products were sequenced with the BigDye Terminator v3.1 Cycle Sequencing kit on a 3730xl DNA Analyzer (both Applied Biosystems). Sequence reads were analyzed with the Sequence Pilot software v4.2.2, Seqpatient module (JSI medical systems, GmbH). The identified novel *DCLRE1C* variant has been uploaded onto the LOVD mutation database.

### Generation of the Minigene Construct

Genomic DNA from both control and patient-derived fibroblasts was isolated using the GenElute Mammalian Genomic DNA Miniprep Kit (Sigma-Aldrich) according to the manufacturer’s instructions. The region covering the mutation and flanking exons were PCR amplified (Q5 polymerase, NEB) using primers containing NotI and XhoI restriction sites. These inserts were first subcloned into the pCR™-Blunt plasmid using the Zero Blunt™ PCR Cloning Kit (ThermoFisher Scientific) by blunt-end cloning. A restriction digest of both the insert-containing pCR™-Blunt plasmids and the pSpliceExpress recipient plasmid was performed using XhoI-HF and NotI-HF restriction enzymes (both NEB). The cut pSpliceExpress was first dephosphorylated using alkaline phosphatase (Roche) according to the manufacturer’s instructions. The digested products were ligated using the Mighty Mix DNA ligation kit (Takara Bio) into the pSpliceExpress plasmid by sticky-end cloning. The ligated products were used to transform chemocompetent *E. coli* (C2987, NEB) after which colony PCR on a sufficient number of selected clones was performed to identify transformants. Correct clones were grown in LB medium containing ampicillin for further selection and expansion of transformants. Plasmid DNA of correct clones was extracted through Midiprep (Qiagen) and finally submitted to Sanger sequencing in order to verify the insertion of the correct sequence derived from control and patient DNA.

Primers: Fwd_DCLRE1C-minigene-XhoI: TAAGCACTCGAGTTTCTACCCGAGGGAAGAGGT; Rev_DCLRE1C-minigene-NotI: AAGAAAAACAAGCTCTGTATGAACTCTCTCC.

### Transfection of HEK Cells and RT-PCR of RNA Transcripts

HEK cells were transfected with 1 µg of the minigene-containing pSpliceExpress vectors using the XtremeGene HP DNA transfection reagents (Roche), according to the manufacturer’s instructions. After 48 hours, total RNA was isolated using Qiazol and the RNeasy micro kit with on-column DNA digestion using the RNase-free DNase set (all Qiagen). Next, total RNA was reversely transcribed using the iScript Advanced cDNA synthesis kit (BioRad) according to the manufacturer’s instructions. Finally, cDNA was purified using MinElute PCR purification columns (Qiagen) and subjected to Sanger sequencing for analysis of alternative splicing events.

### RT-PCR and Western Blotting for Identification of the Truncated Transcripts and Protein

Total RNA was isolated from both control and patient-derived fibroblasts as described above. cDNA was synthesized and RT-PCR was performed using the Taq polymerase with the standard Taq buffer kit (NEB). For western blotting, whole cell lysates were first lysed and denatured in Laemmli buffer in the presence of β-mercaptoethanol. Denatured lysates were loaded on a precast Mini-PROTEAN 10% gel (BioRad) for PAGE before transfer onto nitrocellulose membranes. Next, blocking of the membrane was performed using a 5% non-fat dry milk solution before immunoblotting with anti-human beta-ACTIN (Cell Signaling Technologies) and anti-human ARTEMIS (Biolegend) antibodies, according to the manufacturer’s instructions. Finally, blots were stained using a secondary anti-mouse HRP-linked IgG antibody (Bioké) before visualization using the SuperSignal™ West Dura Extended Duration Substrate (ThermoFisher Scientific) on the ChemiDoc imaging system (BioRad).

Primers:Fwd_DCLRE1C-exon1: CAGATGGCCGAGTATCCAAC; Rev_DCLRE1C-exon6: CTTGCGCCAATCTGAAGTCT; Fwd_DCLRE1C-exon6: ATGGAGCTTCTGCACTCC; Rev_DCLRE1C-exon11: AAGAAAAACAAGCTCTGTATGAACTCTCTCC.

### V(D)J Recombination Assay

V(D)J recombination assays were performed as described by Pannicke et al. (39). Briefly, fibroblasts were transfected using the Amaxa human dermal fibroblast nucleofector kit (Lonza). Transfections were performed using 1.2 µg pcWTRAG1, 1.8 µg pcWTRAG2, 8 µg pMACS11-19VDJ substrate or pMACS11-19Flip and 1 µg pcDNA6/ArtWT-myc-His plasmids. After 48 hours, cells were harvested and analyzed by flow cytometry for identification of EGFP expression.

### G0 Micronucleus Assay

The protocol of Słonina et al. (40) was used with some minor modifications. Briefly, fibroblasts were grown to confluency and irradiated in the G0 phase of the cell cycle with different doses of 220 kV X-rays (0.5 and 1 Gy), followed by the addition of cytochalasin B to block cytokinesis. Cells were harvested after 96 hours and were subsequently fixed with a methanol/acetic acid solution. Slides were stained with acridine orange. Micronuclei were counted in 1,000 binuclear cells per condition. Cells were scored according to standard criteria, as described by Fenech et al. (41)

### Statistical Analysis

Two-tailed paired t-tests were performed using the GraphPad Prism software, version 8.0.1 for Windows (San Diego, California, USA, www.graphpad.com).

## Results

### Genetic Analyses of a T^-^B^-^NK^+^ SCID Patient Reveals a Novel Homozygous Non-Coding c.465-1G>C Variant in the *DCLRE1C* Gene

**Figure 1:**
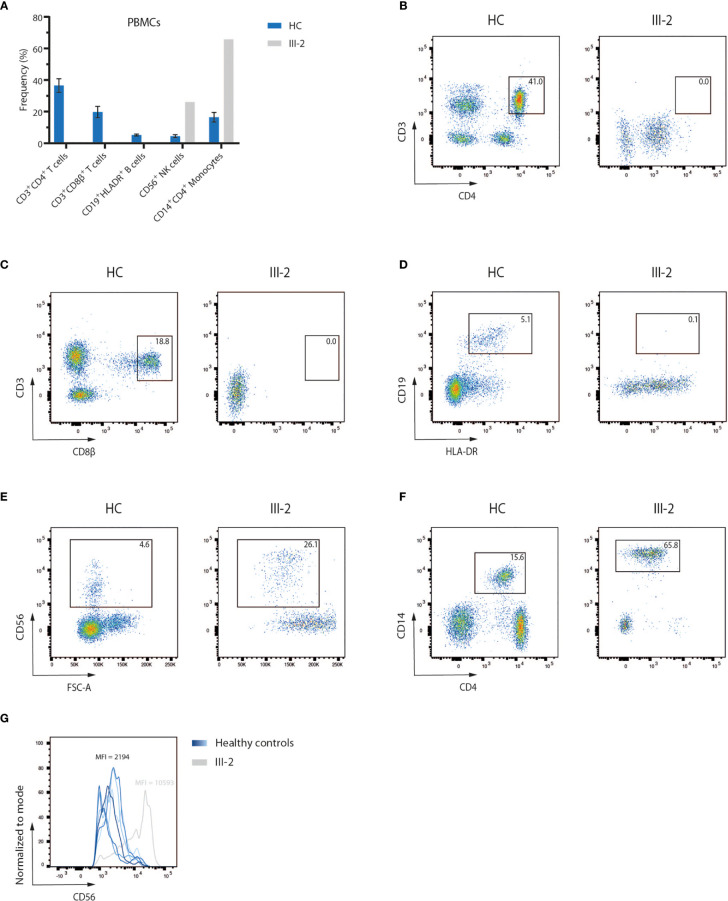
Flow cytometry analysis of PBMCs of a SCID patient shows a lack of T and B lymphocytes **(A)** Flow cytometry analysis of PBMCs of six healthy children and the index patient (III-2). Bars indicate the mean of the corresponding populations with error bars as the standard deviation. HC, Healthy Control; PBMC, Peripheral Blood Mononuclear Cells. **(B–F)** Pseudocolor plots of CD3^+^CD4^+^ and CD3^+^CD8β^+^ T cells, CD19^+^HLADR^+^ B cells, CD56^+^ NK cells and CD14^+^CD4^+^ monocytes in both a representative healthy control and the index patient. Numbers indicate the percentage of the gated populations. HC, Healthy Control. **(G)** Flow cytometry analysis of the CD56 expression level on peripheral NK cells, corresponding to the pseudocolor plots in **(E)**. Numbers indicate the geometric mean fluorescence intensity (MFI) of CD56 expression.

**Figure 2:**
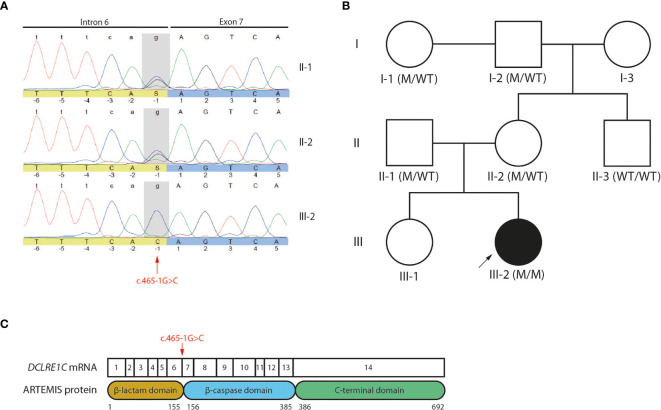
Genetic analyses reveals a novel c.465-1G>C non-coding variant of the *DCLRE1C* gene **(A)** Sanger electropherogram of both the index patient and parents covering the region of the c.465-1G>C variant in the *DCLRE1C* gene. The location of the novel variant is indicated by the red arrow. **(B)** Pedigree of the affected family showing the prevalence of the variant across multiple generations. The index patient is depicted as a black circle and indicated by the black arrow. The genotype of every available family member is denoted. M, mutation; WT, wild-type. **(C)** Schematic representation of the canonical *DCLRE1C* transcript and the canonical ARTEMIS protein. The *DCLRE1C* transcript contains 14 coding exons. The ARTEMIS protein contains 3 protein domains consisting of 692 amino acids. The location of the variant is indicated by the red arrow.

A 4-month-old infant (III-2) was admitted to the hospital with chronic diarrhea and failure to thrive because of decreased intake caused by recurrent aphthae located in the tonsils and pharynx. The index patient did not have any signs of microcephaly, seizure disorder or developmental delay but showed a rash on mouth and cheeks shortly after birth. Furthermore, despite standard rotavirus vaccination, analysis of the stool showed a chronic rotavirus infection. Moreover, imaging of the thorax revealed a rudimentary thymus, indicating reduced thymic function (data not shown). Analysis of the Peripheral Blood Mononuclear Cells (PBMCs) of the index patient showed a severe lymphopenia in comparison to 6 healthy controls. No CD3^+^CD4^+^ (**Figures 1A, B** **)** and CD3^+^CD8β^+^ (**Figures 1A, C** **)** T cells and no CD19^+^HLA-DR^+^ B cells (**Figures 1A, D** **)** could be detected. In contrast, CD56^+^ NK cells (**Figures 1A, E** **)** and CD14^+^CD4^+^ monocytes (**Figures 1A, F** **)** were present, suggesting a T^-^B^-^NK^+^ SCID phenotype. In addition, no T cell receptor excision circles (TRECs) were detected (data not shown). Interestingly, the frequency of CD56^bright^ NK cells was increased (**Figure 1G**) which is frequently observed in patients having mutations in one of the key players of V(D)J recombination (32). To identify the underlying mutation that drives the pathogenesis in this patient, a targeted panel of candidate genes associated with T^-^B^-^NK^+^ SCID was analyzed by NGS. This revealed a homozygous c.465-1G>C mutation at the 3’ end of intron 6 of the *DCLRE1C* gene which was confirmed by Sanger sequencing (**Figure 2A**). Further analysis of both non-consanguineous Caucasian parents (II-1 and II-2) confirmed a heterozygous carrier status of the mutation (**Figure 2A**). Both parents did not have any history of immune deficient-related problems, consistent with the autosomal recessive features of ARTEMIS-SCID and as shown by PBMC analysis compared to an adult healthy control (**Supplemental Figure 1A**). Also, the intensity of CD56 expression on the NK cells of both family members was comparable to that of adult healthy control NK cells, indicating no bias towards a CD56^bright^ phenotype in these heterozygous carriers (**Supplemental Figure 1B**). In addition, familial segregation analysis showed that the genetic trait was conserved across multiple generations in a recessive fashion, in accordance with the mendelian inheritance pattern seen in monogenic SCID (**Figure 2B**). This novel c.465-1G>C non-coding mutation alters the AG splice acceptor site at the 3’ end of intron 6 and might therefore have a direct impact on the canonical splicing pattern of the *DCLRE1C* transcript. Consequently, we hypothesized that the c.465-1G>C nucleotide substitution of the splice acceptor site might therefore induce loss of downstream protein domains which are essential for ARTEMIS function (42, 43) (**Figure 2C**), thereby driving the observed phenotype and pathogenicity. Splicing mutations of *DCLRE1C* have been previously reported but these were, in contrast to our patient with a severe early onset T^-^B^-^NK^+^ phenotype, characterized as hypomorphic with a later disease onset and both T and B cells present (44–46). Moreover, the c.465-1G>C variant found in our patient is predicted to affect all 9 protein-coding RNA transcripts of the *DCLRE1C* gene and might therefore severely impact the function of all 4 ARTEMIS protein isoforms (**Supplemental Figure 2**). The patient received, after mild conditioning using treosulfan, fludarabine and antithymocyte globulin (ATG), a haplo-identical stem cell transplant from the older sibling at the age of 6 months with good engraftment and immune reconstitution and without the need for Ig substitution 3 years post-transplant.

### Analysis of the Splicing Pattern Reveals the Employment of an Alternative Cryptic Splice Acceptor Site That Results in a Frameshift and PTC

**Figure 3:**
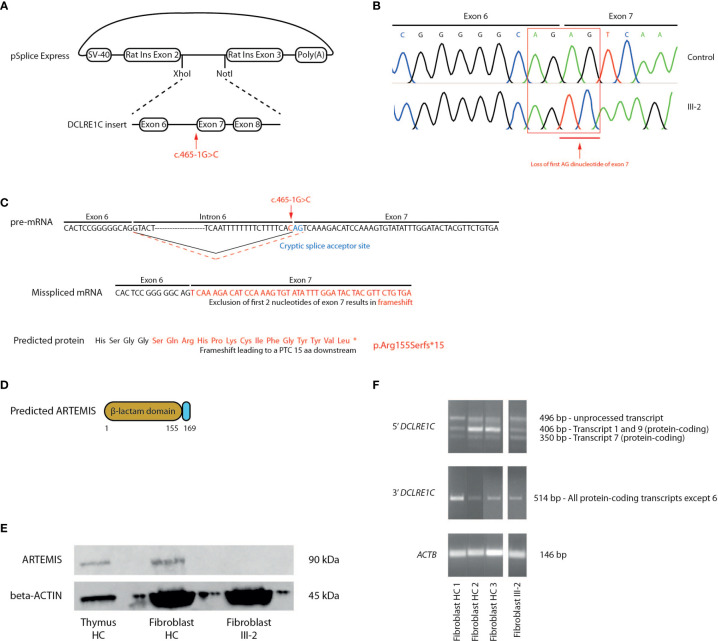
Molecular study of the *DCLRE1C* gene and ARTEMIS protein reveals an alternative splicing mechanism leading to a frameshift mutation and a PTC. **(A)** Schematic representation of the generation of the pSplice Express vector containing the patient *DCLRE1C* minigene with the c.465-1G>C variant. The minigene containing exons 6, 7, 8 and corresponding introns is cloned in between the two rat insulin exons by XhoI and NotI compatible overhangs. The location of the variant is indicated by the red arrow. **(B)** cDNA Sanger sequencing of the minigenes of both control and patient, expressed by host HEK cells. Sanger electropherograms of both minigenes are depicted and the loss of the AG dinucleotide at the start of exon 7 is indicated by the red arrow. **(C)** Schematic representation of the pre-mRNA, mRNA and ARTEMIS protein containing the altered sequence resulting from the alternative splicing. On the pre-mRNA level, the new cryptic splice acceptor site in exon 7 is depicted in blue. The canonical splicing pattern is shown by a solid black line whereas the alternative splicing mechanism is indicated by a dashed red line. The location of the c.465-1G>C variant is depicted in red. On the mRNA and protein level, the altered sequence that results in a frameshift and a PTC is shown in red. The PTC is depicted as an asterisk on the truncated ARTEMIS protein (*). **(D)** Schematic representation of the truncated ARTEMIS protein, as predicted by the alternative splicing event. **(E)** Western blot analysis for the ARTEMIS protein (upper panel) and for beta-ACTIN (lower panel) on total thymus, HC fibroblast and patient fibroblast lysates (n=3). HC, Healthy Control. **(F)** RT-PCR of *DCLRE1C* cDNA of 3 independent fibroblast controls and patient fibroblasts. The upper panel shows the 5’ RT-PCR of *DCLRE1C* cDNA, with 3 bands each representing a different *DCLRE1C* transcript corresponding to the transcripts shown in **Supplemental Figure 2**. The middle panel shows the 3’ RT-PCR of *DCLRE1C* cDNA. The lower panel depicts the RT-PCR of *ACTB* as a positive control. HC, Healthy Control; PTC, Premature Termination Codon.

To demonstrate that the novel c.465-1G>C variant affects splicing of the *DCLRE1C* transcript and therefore might be responsible for the observed pathogenesis in this T^-^B^-^NK^+^ SCID patient, we performed a splicing reporter assay by cloning a minigene, containing the c.465-1G>C mutated intron 6 from the patient and its flanking exons, into the pSpliceExpress vector (47) **(** **Figure 3A** **).** Here, we used the exact same genomic region of healthy fibroblasts to generate a vector in parallel that served as a control. The minigene is placed in between 2 rat insulin exons to allow the unambiguous identification of transcripts solely expressed from this pSpliceExpress vector. After transfection of Human Embryonic Kidney (HEK) cells, reverse transcription-polymerase chain reaction (RT-PCR) allows to amplify these alternative transcripts that might differ in size. Although gel electrophoresis was unable to discern a difference in size between patient and control (data not shown), Sanger sequencing of this amplicon revealed the deletion of the first AG dinucleotide at the start of exon 7 which thus has been used as an alternative cryptic splice acceptor site instead of the mutated site in intron 6 **(** **Figure 3B**). This alternative splicing mechanism has important implications since loss of this AG dinucleotide results in a frameshift that leads to a PTC in exon 7, 15 amino acids downstream of the mutated Arg155 on all *DCLRE1C* transcripts and therefore results in a p.Arg155Serfs*15 mutation at the protein level **(** **Figure 3C** **).** This variant might severely impact ARTEMIS function as the β-caspase and C-terminal domain of the full-length protein are predicted to be absent **(** **Figure 3D** **).** To validate if this PTC indeed impacts the generation of full-length ARTEMIS protein, we performed a western blot analysis for ARTEMIS using a monoclonal antibody that was raised against a part of the protein that is downstream of the PTC. While the ARTEMIS protein was clearly detected in thymocytes and fibroblasts from healthy individuals, no ARTEMIS protein could be detected in the index patient, consistent with the introduction of a PTC **(** **Figure 3E** **).** We also studied the impact of the c.465-1G>C variant on mRNA stability. Using RT-PCR on cDNA from patient-derived fibroblasts, we were able to detect both 5’ and 3’ amplicons, showing that the truncated *DCLRE1C* transcripts are not degraded by non-sense mediated decay (NMD) *in vivo* **(** **Figure 3F** **).** Together, these findings suggest that the c.465-1G>C variant is responsible for the SCID phenotype through the generation of a misspliced *DCLRE1C* mRNA that contains a PTC resulting in a truncated ARTEMIS protein.

### The Truncated ARTEMIS Protein Shows a Severe Loss of V(D)J Recombination Activity

**Figure 4:**
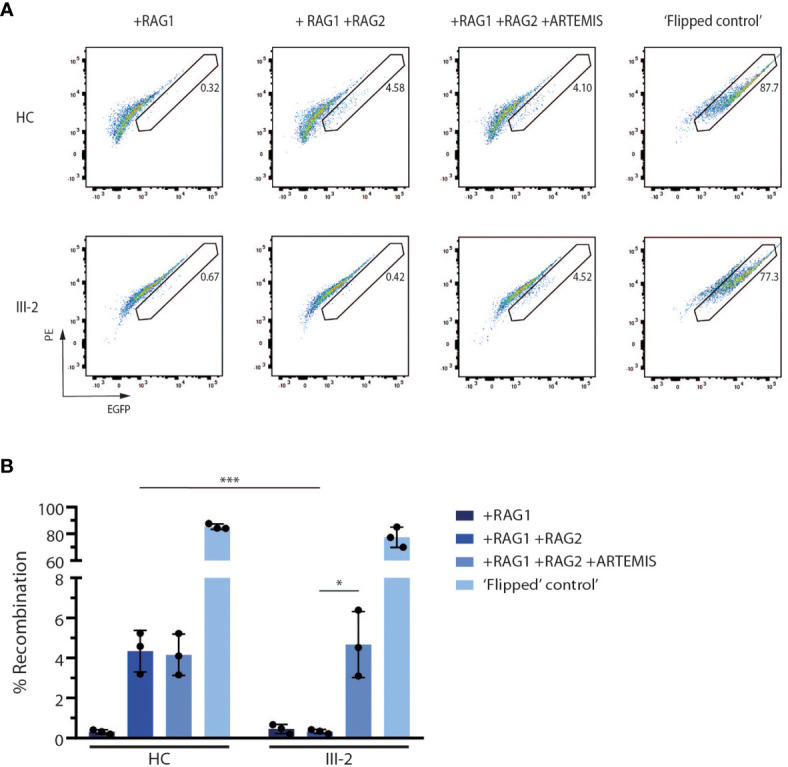
*In vitro* V(D)J recombination assay shows the inability of the truncated ARTEMIS protein to contribute to V(D)J recombination. **(A)** Flow cytometry analysis of the EGFP expression of transfected healthy control and patient fibroblasts with the corresponding constructs as indicated above the pseudocolor plots. EGFP expression is a direct read-out of V(D)J recombination of the substrate. Numbers indicate the EGFP^+^ percentage of the corresponding gated population. As a positive control, recombined substrates are provided (‘Flipped control’). Plots shown are representative for all replicates (n=3). HC, Healthy Control. **(B)** Graph showing the EGFP^+^ percentage of transfected control and SCID fibroblasts with the corresponding constructs. Bars indicate the mean of the 3 independent experiments that are shown by the individual data points and error bars indicating the standard deviation (n=3). ***indicates p < 0.001 and *indicates p < 0.05 resulting from a two-tailed paired t-test. HC, Healthy Control.

Since ARTEMIS is indispensable for V(D)J recombination during early T and B lymphopoiesis, we assessed the potential of the predicted truncated ARTEMIS protein from the index patient to support V(D)J recombination. Here, a V(D)J recombination substrate is co-transfected with RAG1- and RAG2-expressing constructs in both healthy and patient-derived dermal fibroblasts as previously described (39). Upon successful recombination, which is only possible if both RAG expression cassettes are provided and when an endogenously expressed functional ARTEMIS protein is present, an EGFP cassette is cut out of the recombination signal sequences (RSSs) and inverted in the correct orientation which allows the cassette to be expressed under the control of the SV40 promotor. Consequently, the level of EGFP expression is a read-out for the V(D)J recombination activity which can be detected by flow cytometry. Since ARTEMIS is not provided alongside the substrate and the RAG1 and RAG2 constructs, the activity of the endogenously expressed ARTEMIS protein in the patient-derived fibroblasts can be evaluated *in vitro*. As anticipated, the index patient-derived fibroblasts failed to express EGFP when only RAG1 and RAG2 were transfected, in contrast to healthy control fibroblasts, clearly illustrating the inability of the predicted truncated ARTEMIS protein to support V(D)J recombination **(** **Figures 4A, B** **).** Co-transfection of an ARTEMIS-expressing construct in the index patient-derived fibroblasts rescued V(D)J recombination in these transfected cells **(** **Figures 4A, B** **).** These findings clearly illustrate that the novel *DCLRE1C* variant of the index patient results in a deficient V(D)J recombination machinery due to a severe loss of ARTEMIS activity.

### Patient-Derived Fibroblasts Display a Strong Reduction in DNA Repair Activity Using a Micronucleus Assay

**Figure 5:**
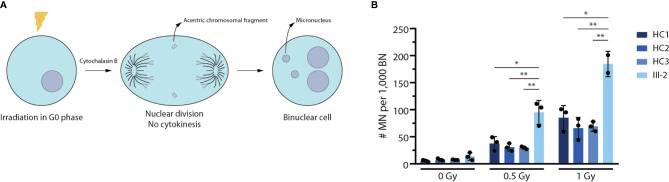
Truncated patient-derived ARTEMIS protein is unable to contribute to DNA repair in a G0 micronucleus assay. **(A)** Schematic representation of the G0 micronucleus assay. Fibroblasts are irradiated at the G0 phase of the cell cycle before addition of cytochalasin B to block cytokinesis. Upon nuclear division, the micronuclei (MN) resulting from acentric chromosomal fragments can be counted relative to the amount of binucleate cells (BN). **(B)** Graph showing the relative abundance of micronuclei compared to binuclei in 3 independent healthy control and patient fibroblast lines. Cells are irradiated with increasing intensities of Gray (Gy). Bars indicate the mean of the 3 independent experiments that are shown by the individual data points and error bars indicating the standard deviation (n=3). **indicates p < 0.01 and * indicates p < 0.05 resulting from a two-tailed paired t-test. HC, Healthy Control.

Since ARTEMIS is an essential NHEJ factor that also plays a prominent role in DNA damage repair during cell division to maintain genome stability (48–50), we performed a G0 micronucleus assay on both healthy control and patient-derived fibroblasts. In this assay, fibroblasts are irradiated using different dosages of 220 kV X-rays after which their DNA repair potential is assessed by counting the number of micronuclei. These radiation-induced micronuclei mainly arise from acentric chromosomal fragments that lag behind during anaphase and are scored in binucleate daughter cells resulting from cytokinesis block (**Figure 5A**). Here, we observed a significantly higher number of micronuclei in patient-derived fibroblasts compared to 3 independent healthy controls **(** **Figure 5B** **)**, indicating that there is an absence of functional ARTEMIS protein in the index patient-derived fibroblasts to contribute to proper DNA repair upon irradiation using different dosages. In conclusion, this assay shows that the predicted truncated ARTEMIS protein from the index patient inadequately contributes to DNA repair prior to cell division during the cell cycle.

## Discussion

The recent advancements in diagnostic tools have significantly extended the spectrum of disease phenotypes, resulting in an increased need for personalized medicine. This correlates with the huge increase in sequencing data that permits to identify the underlying molecular mechanisms. Consequently, appropriate functional interpretation of these genotypes, that explain corresponding phenotypes, therefore becomes increasingly important since a huge number of non-pathogenic variants or variants of unknown significance are identified. This is further complicated by the fact that disease-causing mutations can occur in both coding and non-coding parts of the genome and *in silico* prediction tools are insufficient in most cases. Here, we used a targeted NGS panel to identify the underlying genotype for a SCID patient that lacked both T and B cells. This allowed us to identify a novel non-coding mutation in the *DCLRE1C* gene for which we employed and designed several functional assays to validate its disease-causing mechanism.

Until 10 years ago, several genetic approaches and in particular Sanger sequencing was widely used to establish a correlation between the observed phenotype and the underlying genotype (51). The main disadvantage of this approach is that it is only useful if the patient phenotype is unambiguous and can only be linked to a specific gene, but this is rarely the case. Therefore, the development of NGS technologies such as targeted panel-based sequencing, whole exome sequencing (WES), whole genome sequencing (WGS) and RNA sequencing (RNA-seq) allowed for massive parallel sequencing of genetic material. These approaches enabled researchers and clinicians to identify disease-causing variants using increasingly larger datasets. Panel-based sequencing is a cost-efficient method and relies on a panel of known genes that cause the disease of interest. Consequently, this approach is highly dependent on the list of genes that is interrogated and can therefore not detect variants in genes that are not included and that thus are not yet linked to the disease being studied. In contrast, WES can detect variants throughout the whole protein-coding exome but can suffer from insufficient coverage or bias towards some regions because of the enrichment or capture step (52). Nevertheless, WES is often the method of choice for cases where the variants are located in the coding part of the genome and was for instance also successfully applied to identify variants implicated in Common Variable Immune Deficiency (CVID), another type of PID (53). Variants located in non-coding regions such as introns, intergenic regions and regulatory sequences (promotors, enhancers) are increasingly being brought to the attention of geneticists to be pathogenic. However, intronic regions can often not be detected by WES and instead require WGS for their identification although some WES approaches investigate intronic sequences up to 20 nucleotides and thus include canonical splice sites. WGS is also superior to WES for detecting pathogenic structural variants and copy number variations (CNVs) since these genomic alterations would have to be located completely within exons in order to be detected by WES (54). Because of the reasons mentioned above, WGS has already been used for the diagnosis of PID (55, 56). Apart from being the most expensive NGS method, WGS detects significantly more variants which complicates the identification of the causal variant implicated in the disease of interest. Conclusively, this highlights the need for functional validation experiments.

The targeted NGS that we performed on the genetic material of the index patient allowed us to identify a novel homozygous c.465-1G>C non-coding variant in the *DCLRE1C* gene that alters the evolutionary conserved splice acceptor site of intron 6 and might therefore impact the canonical splicing of the *DCLRE1C* pre-mRNA. We were able to validate this prediction by performing a minigene splicing reporter assay which allowed us to precisely identify the alternative cryptic AG splice acceptor site at the start of exon 7. The altered splicing resulted in a frameshift and introduced a PTC 15 amino acids downstream, further referred to as p.Arg155Serfs*15. Importantly, this exon is affected in all *DCLRE1C* transcripts that have been described and validation of this altered splicing event therefore allows us to predict that all ARTEMIS isoforms will be affected by this novel variant. Moreover, this c.465-1G>C variant is the first non-coding splice site mutation of the *DCLRE1C* gene to cause a severe SCID phenotype as opposed to previously reported hypomorphic splice mutations of the *DCLRE1C* gene that resulted in intronic inclusions and exon skipping rather than a severe loss of the full-length ARTEMIS protein (45, 46). Our study thus illustrates that genetic analysis of SCID patients in both coding and non-coding regions, combined with assessment of the functional impact of these variants, could be important in determining appropriate treatment regimens for each individual patient. This could for instance involve avoiding the use of DNA-damaging conditioning agents or predictions on the feasibility of gene therapy.

The formal demonstration of the altered splicing and the identification of the resulting PTC allowed us to predict that a large part of the β-caspase domain should be absent from the ARTEMIS protein in the index patient, in addition to the entire downstream C-terminal domain. Western blot analysis indeed validated the absence of a full-length ARTEMIS protein in the index patient-derived fibroblasts, thereby confirming the predicted alternative splicing and establishing a clear connection with the severe phenotype that was observed in this SCID patient. Although we could not formally demonstrate the presence of the N-terminal part of ARTEMIS upstream of the PTC due to the lack of an appropriate antibody that is capable of targeting this region, we believe that it is highly likely that a truncated ARTEMIS protein is generated by the cells from this patient since we could still detect RNA transcripts that correspond to both the 5’ and 3’ end of the identified mutation. Thus, although mammalian cells have established mechanisms to degrade abnormal transcripts, such as NMD, in order to avoid the translation of gain-of-function (GOF) transcripts that might cause disease, we assume that the NMD pathway is not targeting these newly generated transcripts (57). During splicing of pre-mRNA, exon-junction complexes (EJCs) are being deposited by the spliceosome 20-24 nucleotides upstream of exon-exon junctions (58–60). These complexes act as a safeguard mechanism to ensure that the NMD pathway is activated if the ribosome is unable to physically remove these EJCs during translation. This is indeed the case when the distance between the PTC and the final exon-exon junction is more than 50-55 nucleotides and therefore might result in disease (61–63). Although current models predict that this mutation should activate NMD since the PTC is located more than 50-55 nucleotides away from the final exon-exon junction, we were still able to detect the *DCLRE1C* transcript at both the 5’ and 3’ ends. Therefore, since NMD presumably fails to degrade the *DCLRE1C* transcripts, we assume that not only the position of the PTC determines activation of the NMD pathway as previously also described for mutations in the first exon of the human *HBB* gene (64).

Since *in silico* prediction tools are insufficient to correlate the causality of a new variant to the observed phenotype, and to formally demonstrate that the truncated ARTEMIS protein in the index patient is indeed non-functional, we functionally studied both its recombination and DNA repair potential using well-designed and specific assays. V(D)J recombination is an essential mechanism during the development of early T and B lymphocytes for the generation of a highly diverse repertoire of antigen receptors (65). The inability of the truncated ARTEMIS protein to support V(D)J recombination was clearly demonstrated using an appropriate reporter assay, thereby unambiguously clarifying the lack of mature T and B lymphocytes in the peripheral blood of this patient. The observation that reporter gene expression was rescued in the patient-derived fibroblasts upon introduction of a wild-type ARTEMIS-expressing construct directly confirms that the impaired V(D)J recombination is solely due to the novel *DCLRE1C* variant and not due to any other potential variant in the genome of this patient. This method is therefore highly specific and easy to perform in patient-derived cells for addressing the functional consequences of any genetic variant involved in V(D)J recombination, apart from *RAG1* and *RAG2* variants (39).

ARTEMIS is not only crucial for V(D)J recombination of TCR and Ig loci. It is equally important for DNA repair during both the G1/S and S/G2 phases of the cell cycle to maintain genomic stability (66). DNA repair is a strictly regulated process and occurs through both NHEJ and homologous recombination (HR) (67). During the G1 phase, ARTEMIS mediates resection-dependent NHEJ since this pathway, which accounts for 20% of DSB repairs at this checkpoint, relies on the endonucleolytic activity of ARTEMIS to open the hairpin intermediates (68). During the G1/S checkpoint of the cell cycle, ARTEMIS inhibits p53 activation to ensure progression beyond this checkpoint since p53 is known to induce cell cycle arrest of cells that display genomic abnormalities during G1 (69). Furthermore, ARTEMIS is also involved during HR at the late S/G2 phase of the cell cycle since HR also relies on DNA end resection to remove lesions and secondary structures that result from DNA replication (70). Finally, after DNA repair during late S/G2, the inhibition of ARTEMIS is important for the recovery at the G2/M phase of the cell cycle through downregulation of the negative cell cycle regulator CDK1 (71). As a result, ARTEMIS-SCID is often referred to as radiosensitive (RS)-SCID since irradiation of cells that harbor defects in the *DCLRE1C* gene display defective DNA damage repair and therefore might contribute to genomic instability and, if malignant, tumorigenesis (72). To address the residual DNA repair activity associated with this predicted truncated protein, we performed an adapted G0 micronucleus assay since this method can identify micronuclei that result from acentric chromosomal fragments during cell division upon irradiation. Indeed, we observed a significantly higher number of micronuclei compared to 3 independent healthy controls, confirming the severely reduced capability of the predicted truncated ARTEMIS protein of the index patient to contribute to DNA repair and further confirming the direct pathogenic impact of this novel mutation on protein function.

In conclusion, we have functionally validated the impact of a novel non-coding variant in the *DCLRE1C* gene at both the genetic and downstream protein level. As such, our study is a clear example of how experimental validation of novel variants, identified through NGS-based approaches, can provide unambiguous insights into the precise molecular mechanisms that drive pathogenesis. As such, our findings can serve as a framework for future studies that aim to functionally unravel new genetic variants. This is important to facilitate the diagnosis of patients with immune disorders and to implement appropriate therapies tailored to the immune deficient patient.

## Data Availability Statement

The original contributions presented in the study are included in the article/**Supplementary Material**. Further inquiries can be directed to the corresponding author.

## Ethics Statement

The studies involving human participants were reviewed and approved by Medical Ethical Commission of Ghent University Hospital (Belgium). Written informed consent to participate in this study was provided by the participants’ legal guardian/next of kin.

## Author Contributions

SS, MDB, UP and EBe performed experiments and analyses. EBa, VB and FH performed and analyzed data acquisition. GL and BV provided critical reagents. SS, TT, KS and AV designed the research. SS and TT wrote the manuscript. All authors contributed to the article and approved the submitted version.

## Funding

This work was supported by the Fund for Scientific Research Flanders (FWO, fellowship grant to SS) and grants from the Ghent University Research Fund (GOA and Starting Credit to TT).

## Conflict of Interest

The authors declare that the research was conducted in the absence of any commercial or financial relationships that could be construed as a potential conflict of interest.

## Supplementary Material

Supplementary Figure 1 Flow cytometry of PBMCs of the patient’s family shows no aberrant phenotype compared to an adult healthy control. **(A)** Flow cytometry analysis of T and B lymphocytes in the peripheral blood of the mother (II-2) and maternal grandfather (I-2) compared to an adult healthy control. Numbers indicate the percentage of the gated populations. HC: Healthy Control. **(B)** Flow cytometry analysis of peripheral NK lymphocytes and their CD56 expression level. Numbers indicate the percentage of gated populations. Numbers in histograms indicate the geometric Mean Fluorescence Intensity (MFI) of CD56 expression.

Supplementary Figure 2 All 9 canonical protein-coding *DCLRE1C* transcripts are predicted to be impacted by the c.465-1G>C variant through the generation of an in-frame PTC. The *DCLRE1C* gene encodes for 16 transcripts of which 13 are protein-coding. 4 of these protein-coding transcripts translate into 4 small proteins which are only predicted models by the Ensembl genome browser and the Uniprot repository. The other 9 protein-coding *DCLRE1C* transcripts will encode the 4 main human ARTEMIS protein isoforms detected *in vivo.* Unfilled boxes: untranslated regions (UTRs). Filled boxes: coding exons. Connecting lines: introns. The 3’ UTR is not depicted since this region is rather large to include in this figure. The location of the variant is indicated by the red arrow.
