# Supplementary figures and images for: A Novel Non-Coding Variant in DCLRE1C Results in Deregulated Splicing and Induces SCID Through the Generation of a Truncated ARTEMIS Protein That Fails to Support V(D)J Recombination and DNA Damage Repair
Source: Front Immunol. 2021 Jun 17;12:674226. doi: 10.3389/fimmu.2021.674226 (PMC8248492; doi:10.3389/fimmu.2021.674226)

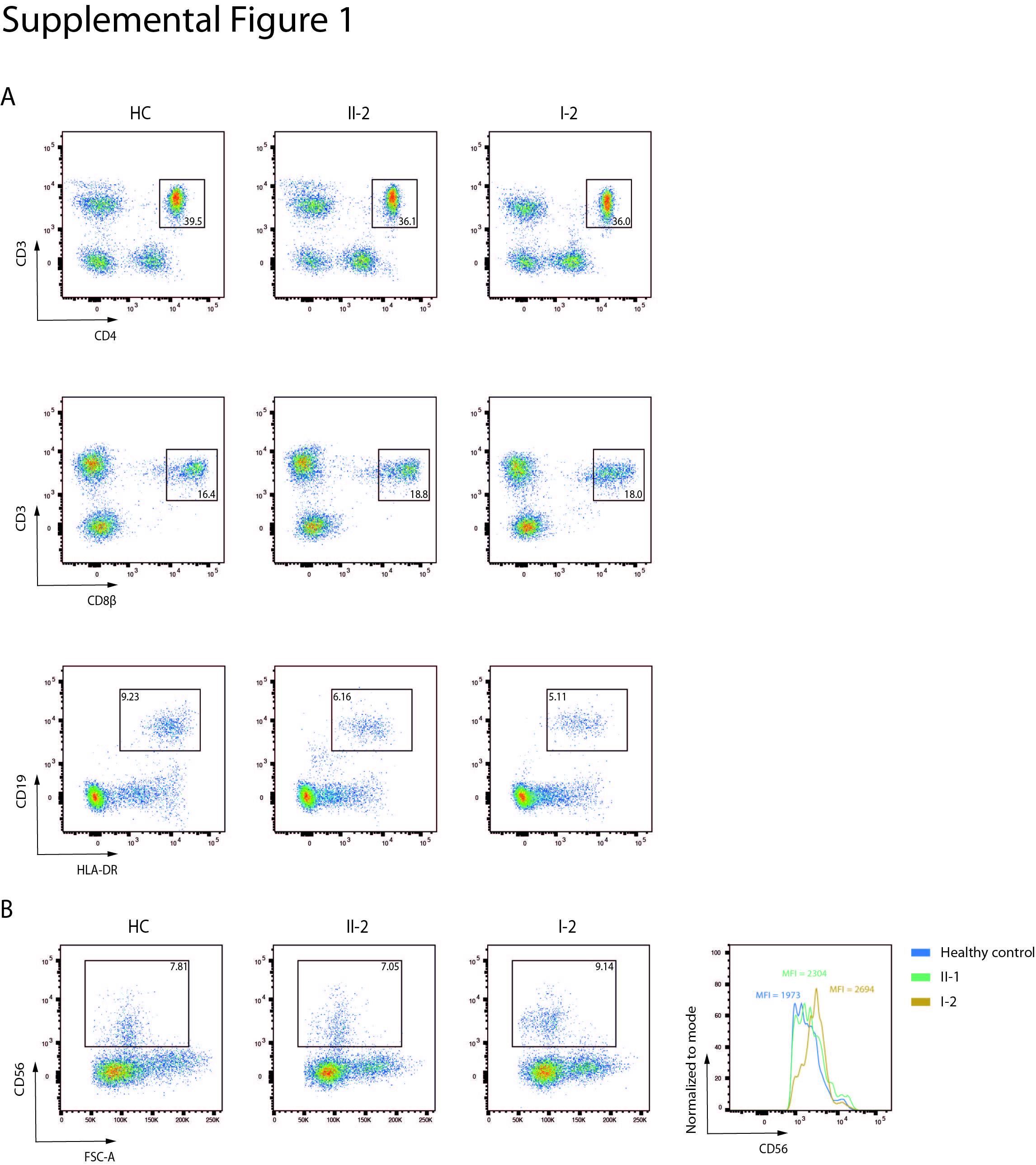

Supplement: Supplementary Figure 1 — Flow cytometry of PBMCs of the patient’s family shows no aberrant phenotype compared to an adult healthy control. (A) Flow cytometry analysis of T and B lymphocytes in the peripheral blood of the mother (II-2) and maternal grandfather (I-2) compared to an adult healthy control. Numbers indicate the percentage of the gated populations. HC: Healthy Control. (B) Flow cytometry analysis of peripheral NK lymphocytes and their CD56 expression level. Numbers indicate the percentage of gated populations. Numbers in histograms indicate the geometric Mean Fluorescence Intensity (MFI) of CD56 expression. [file Image_1.jpeg]

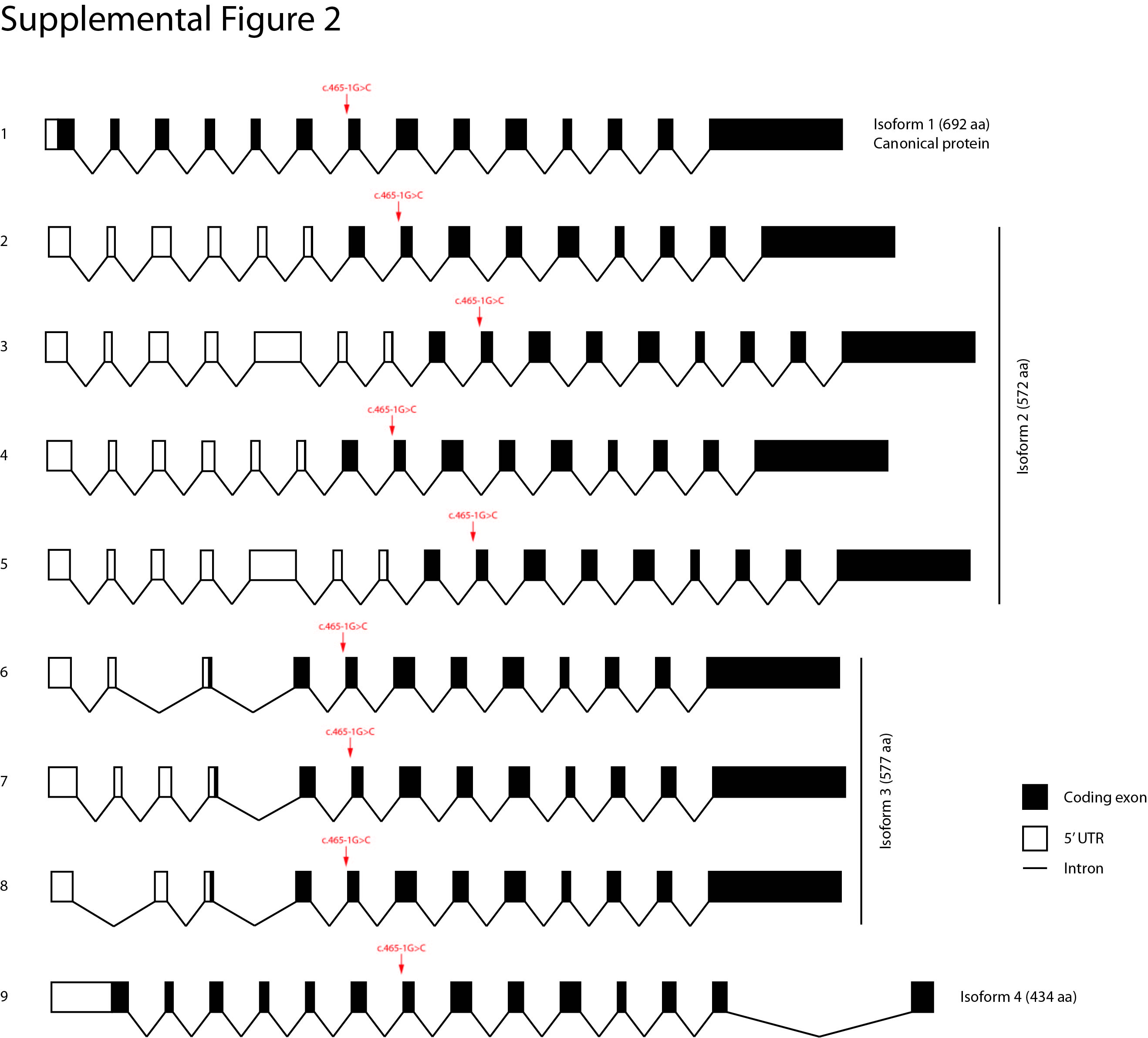

Supplement: Supplementary Figure 2 — All 9 canonical protein-coding DCLRE1C transcripts are predicted to be impacted by the c.465-1G>C variant through the generation of an in-frame PTC. The DCLRE1C gene encodes for 16 transcripts of which 13 are protein-coding. 4 of these protein-coding transcripts translate into 4 small proteins which are only predicted models by the Ensembl genome browser and the Uniprot repository. The other 9 protein-coding DCLRE1C transcripts will encode the 4 main human ARTEMIS protein isoforms detected in vivo. Unfilled boxes: untranslated regions (UTRs). Filled boxes: coding exons. Connecting lines: introns. The 3’ UTR is not depicted since this region is rather large to include in this figure. The location of the variant is indicated by the red arrow. [file Image_2.jpeg]
